# Preparedness of lower-level health facilities and the associated factors for the outpatient primary care of hypertension: Evidence from Tanzanian national survey

**DOI:** 10.1371/journal.pone.0192942

**Published:** 2018-02-15

**Authors:** Deogratius Bintabara, Bonaventura C. T. Mpondo

**Affiliations:** 1 Department of Public Health, College of Health Sciences, The University of Dodoma, Dodoma, Tanzania; 2 Department of Internal Medicine, College of Health Sciences, The University of Dodoma, Dodoma, Tanzania; Makerere University School of Public Health, UGANDA

## Abstract

**Introduction:**

Sub-Saharan Africa is experiencing a rapid rise in the burden of non-communicable diseases in both urban and rural areas. Data on health system preparedness to manage hypertension and other non-communicable diseases remains scarce. This study aimed to assess the preparedness of lower-level health facilities for outpatient primary care of hypertension in Tanzania.

**Methods:**

This study used data from the 2014–2015 Tanzania Service Provision Assessment survey. The facility was considered as prepared for the outpatient primary care of hypertension if reported at least half (≥50%) of the items listed from each of the three domains (staff training and guideline, basic diagnostic equipment, and basic medicines) as identified by World Health Organization-Service Availability and Readiness Assessment manual. Data were analyzed using Stata 14. An unadjusted logistic regression model was used to assess the association between outcome and explanatory variables. All variables with a P value < 0.2 were fitted into the multiple logistic regression models using a 5% significance level.

**Results:**

Out of 725 health facilities involved in the current study, about 68% were public facilities and 73% located in rural settings. Only 28% of the assessed facilities were considered prepared for the outpatient primary care of hypertension. About 9% and 42% of the assessed facilities reported to have at least one trained staff and guidelines for hypertension respectively. In multivariate analysis, private facilities [AOR = 2.7, 95% CI; 1.2–6.1], urban location [AOR = 2.2, 95% CI; 1.2–4.2], health centers [AOR = 5.2, 95% CI; 3.1–8.7] and the performance of routine management meetings [AOR = 2.6, 95% CI; 1.1–5.9] were significantly associated with preparedness for the outpatient primary care of hypertension.

**Conclusion:**

The primary healthcare system in Tanzania is not adequately equipped to cope with the increasing burden of hypertension and other non-communicable diseases. Rural location, public ownership, and absence of routine management meetings were associated with being not prepared. There is a need to strengthen the primary healthcare system in Tanzania for better management of chronic diseases and curb their rising impact on health outcomes.

## Introduction

Currently, non-communicable diseases (NCDs) are the leading cause of adult death worldwide compared to all other causes combined [1]. In low and middle-income countries, it is estimated that up to 80% of all deaths are related to NCDs [1–3]. Despite the fact that infectious diseases are still predominant in Sub-Saharan Africa (SSA), the region is experiencing a rapid rise in the prevalence of NCDs [4–6] and thus now faces a double burden of diseases [7]. Among the NCDs, the prevalence of hypertension and diabetes mellitus has substantially risen in developing countries to become key diseases of public health concern [5,8,9]. Projections show that the burden of NCDs will continue to rise in these countries and significantly contribute to morbidity and mortality by the year 2030 [10,11]. In view of this, the United Nations General Assembly met in 2011 that emphasized on the need to strengthen measures for prevention and control of NCDs in developing countries [12].

Compared to other NCDs, hypertension (HTN) has been reported as a more substantial contributor to morbidity in SSA in which almost a half population already reported to suffer from HTN [13,14]. Also, it has been ranked as the third among causes of disability-adjusted life years (DALYs) [15]. Evidence shows that the prevalence of HTN in developing countries currently is comparable to developed countries [16–18]. Studies in SSA show the prevalence of HTN range between 14% to 69.9% with a median prevalence of 29% [19]. This prevalence is expected to further increase by 60% in developing countries including SSA [17]. Projections show that the number of adults with HTN in SSA will increase from 75 million to 125 million by the year 2025 [20]. Measures for prevention, detection, management, and control of HTN should, therefore, be given high priority in SSA [21].

In Tanzania, like other countries in SSA, HTN has been reported to be the most common NCD [22]. The increased number of hypertensive patients has over-stretched the health care system in the country, thus compromising the quality of services. The Tanzanian government has tried to overcome this challenge by adopting a new strategy for prevention and control [23], increasing the number of health facilities, training more health workers, and increasing supplies of important equipment and medicines. However, there is an uneven distribution of these services by location, managing authority, and type of facility [24–26].

Data regarding the capacity and preparedness of facilities to offer primary care for the outpatient management of HTN and other NCDs remains scarce in Tanzania and the SSA region as a whole. Few studies which have been done in Tanzania and Uganda found low levels of preparedness to offer HTN services despite the rising burden [27,28]. Also, they pointed out that the situation is worse at the lower-level health facilities (health center, dispensaries, and clinics), which are usually located in rural areas and serve about 75% of population. However, these studies analyzed data collected from a single region and therefore lack national representativeness and did not assess the possible factors for preparedness. Therefore, the current study aimed to assess the level of preparedness of lower-level health facilities in Tanzania for the outpatient management of HTN and identify factors associated with preparedness by using data from a national representative sample.

## Materials and methods

### Data source

This study used data from the 2014–2015 Tanzania Service Provision Assessment (TSPA) survey administered by the Ministry of Health and Social Welfare (MoHSW) of Tanzania. This survey was designed to collect information about the availability and readiness of basic health care services at Tanzania health facilities. It assessed the presence and function of components essential for quality service delivery in the areas of child health, maternal and newborn care, family planning (FP), antenatal care (ANC), sexually transmitted infections (STIs), HIV/AIDS, and non-communicable diseases (NCDs) [29].

### Sample size and sampling techniques

The 2014–2015 TSPA survey calculated and randomly sampled a total of 1,200 health facilities out of a sampling frame containing all health facilities (7,102) in Tanzania to be interviewed. The determined sample was designed to provide nationally representative results according to facility type, managing authority and regionally representative results for both the Tanzania mainland and Zanzibar regions. Since facilities were randomly selected from the facility list, oversampling has been done to the regions with small population size and some facilities such as the hospital that exist in fewer numbers than other types of the facility in order to have enough number of those facilities in the sampling domain.

The 2014–2015 TSPA used four main types of data collection tools: a facility inventory questionnaire; a health provider interview questionnaire; observation protocols for ANC, FP, and sick child services; and exit interview questionnaires for ANC and FP clients and for caretakers of sick children whose consultations were observed. The information collected from each questionnaire was stored in a different file [29]. The current study analyzed data from the facility inventory file. For that reason, the unit of analysis remained at the facility level.

In order to answer our research questions, the current study was restricted to lower-level health facilities that reported to offer HTN services. Therefore, facilities such as health centers, dispensaries, and clinics that were open on the day of the interview and agreed to participate were included in this study. Hence, the following facilities were excluded because of not meeting the inclusion criteria; seven refused to participate, four were closed down on the day of the interview, 418 were not providing any HTN service, and 45 were the high-level facility (hospitals). After removing all facilities that did not meet inclusion criteria, only 725 out of 1,200 selected facilities were included in the current study.

### Operational definition

#### Hypertension services

In this study is defined as “ability of the facility to provide either diagnosis or management of uncomplicated case(s) of hypertension”.

#### Preparedness

In this study refers to readiness of health facility to provide primary care for uncomplicated HTN. The facility was considered as “prepared” if reported to have at least a half (score of ≥50%) of the important items in each of the following three domains; staff training and guideline (guideline and staff); diagnostic equipment (blood pressure (BP) apparatus, a stethoscope, a weighing scale, and oxygen concentrator/cylinder); and basic medicine and commodities (angiotensin-converting enzyme (ACE) inhibitors, thiazides, beta blockers and calcium channel blockers), and less than a half (<50%) considered as “not prepared”.

### Measurement of variables

#### Outcome variable

The primary outcome variable in this study was “preparedness” of lower-level health facilities. The preparedness variable was rated as an index categorized into three domains as suggested by the World Health Organization-Service Availability and Readiness Assessment (WHO-SARA) reference manual [30]. The first domain was staff and guidelines which had two indicators: presence of a guideline for HTN management and staff trained to manage HTN; it was categorized as “Yes” for facilities with guidelines and at least one staff who had received training in HTN and “No” otherwise. The second domain was diagnostic equipment which had four indicators: the presence of BP apparatus, stethoscope, weighing scale, and an oxygen concentrator/cylinder. Each equipment indicator was categorized as “Yes” for facilities reporting the presence of the equipment and "No" for facilities reporting the absence of each equipment type. The third domain was medicine and commodities which had four indicators: the availability of ACE inhibitors, thiazides, beta blockers and calcium channel blockers, and each was categorized as “Yes” for facilities reporting the availability of that medicines and “No” for facilities reporting unavailability of that medicine. The responses were aggregated into an index score using the WHO approach to calculate a composite score. The index score was calculated by adding the presence of each indicator, with equal weight given to each of the domains and each of the indicators within the domains. Since the target was 100%, each domain accounted for 33.3% (100%/3) of the index. The percent for each indicator within the domain was equal to 33.3% divided by the number of indicators in that domain. Then, the facility that scores at least half (equivalent to the median value of ≥16.7% and above) in each domain and adding up to the overall of 50% or more were considered as “prepared” for the outpatient management of HTN while those with low than 50% were considered as “not prepared”. The cut-off point used in the current study was also used in previous studies to dichotomize the outcome variable [31–33].

#### Explanatory variables

The facility type was coded as “health center” and “clinic/dispensary”; location was coded as “mainland” and “Zanzibar”; residence was coded as “urban” and “rural”; managing authority was coded as “public” and “private”; routine management meetings were coded as “performed” for facilities conducting routine management meetings and “Not performed” for facilities that reported not having routine management meetings; external supervision was coded as “received” for facilities that reported receiving external supervision and “not received” for facilities that reported not receiving external supervision; external source of revenue was coded as “none”, “government” and “non-government”; HTN guideline was coded as “available” and “not available”; user fees was coded “fixed for all services” and “separate”; training about HTN was coded as “available” and “not available”.

### Data processing and analysis

In order to answer our research questions, our analysis was restricted to lower-level health facilities (health centers, dispensaries, and clinics) that reported to offer HTN services. Therefore, after removal of facilities not meeting these criteria (7 refused, 4 closed down, 4 not reached, 418 not providing HTN services, and 45 were hospital-level facility), only 725 out of 1,200 selected facilities were included.

Data were analyzed using Stata 14 (StataCorp, College Texas). For all the analyses, the “svy” set command in Stata was used to adjust for the complex sampling design used by TSPA survey. All estimates were weighted to correct for non- response and disproportionate sampling.

In the descriptive analysis, categorical variables were summarized using proportions and then presented in tables and graphs. An unadjusted logistic regression model was fitted to assess whether an association existed between the outcome variable “preparedness” and the explanatory (health facility) variables; location, residence, facility type, managing authority, management meeting, external supervision, external source of revenue, type of user fees and acceptability of health insurance. Then, all variables with a *P* value < 0.2 were fitted into the multiple logistic regression models using the stepwise (backward elimination) method to test for the association of each variable with the outcome variable using a 5% significance level. The resulting final model included all the variables that determine the “preparedness”. The *P*-value and the 95% confidence interval (CI) for the odds ratio (OR) were used to confirm the significance of the associations. A *P*-value of less than 0.05 was considered statistically significant.

### Ethical considerations

The current study was based on an analysis of existing public domain (The 2014–2015 TSPA) survey datasets that are freely available online with all identifier information detached. The 2014–2015 TSPA survey was approved by Tanzania’s National Institute for Medical Research (NIMR), the Zanzibar Medical Ethics and Research Committee (ZAMREC) and the Institutional Review Board of ICF International in the USA. The informed consent was requested and obtained from the manager, the person-in-charge of the facility, or the most senior health worker responsible for client services who was present at the facility. The respondents were adequately informed about all relevant aspects of the study, including its aim and interview procedures. Respondents, who accepted their facilities to participate in the study, provided a signed written informed consent.

## Results

### Baseline characteristics of the facilities

Table 1, summarizes the baseline characteristics of the facilities involved in this study. Out of the 1200 facilities involved in the survey, 725 were the lower-level health facilities offering HTN services and therefore were included in this analysis. The majority of the sampled facilities (95%) were in mainland Tanzania compared to Zanzibar. Of the total number of facilities involved in the study, 68% were public facilities of which 73% were located in the rural setting. Most of the facilities (84%) were either clinics or dispensaries.

**Table 1:** Baseline characteristics of the health facilities surveyed during Tanzania SPA 2014–15 (N = 725).

Variable	Number (weighted) (n)	Percentage (%)
**Facility location**		
Mainland	686	95.3
Zanzibar	39	3.7
**Managing authority**		
Public	496	68.5
Private	229	31.5
**Facility type**		
Clinic & dispensary	608	83.9
Health center	117	16.1
**Facility residence**		
Urban	195	26.8
Rural	530	73.2
**Routine management meetings**		
Not performed	142	19.5
Performed	583	80.5
**External supervision**		
Not received	23	3.1
Received	702	96.9
**External source of revenue**		
Government	373	51.4
Other than government	271	37.4
None	81	11.2
**User fees**		
Fixed for all services	438	60.4
Separate for each service	287	39.6
**Guidelines for HTN**		
Not available	558	76.9
Available	167	23.1
**Staff trained about HTN**		
Not available	636	87.7
Available	89	12.3

### Facility preparedness for the outpatient primary care of hypertension

**Fig 1:**
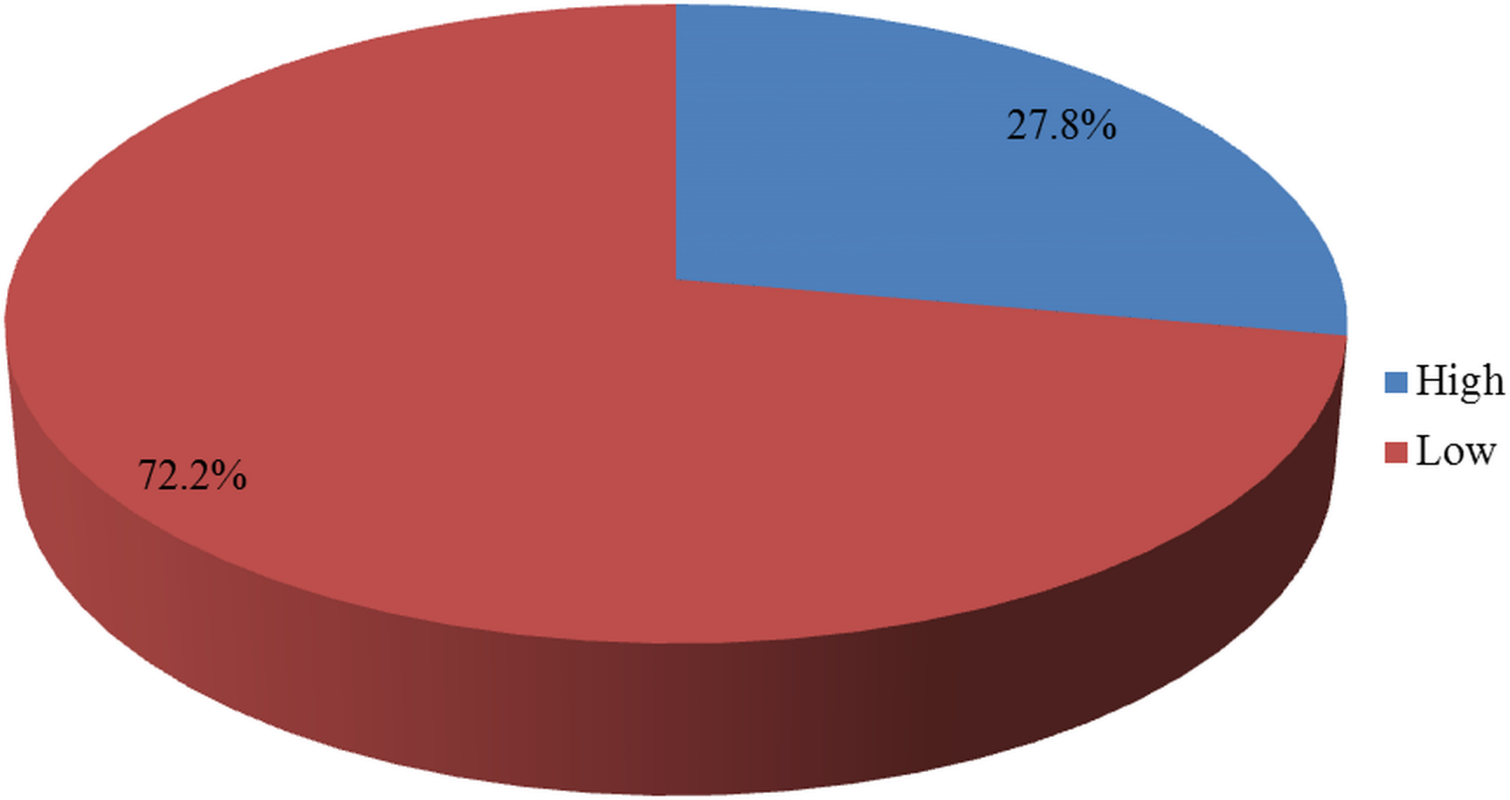
Percentage distribution of facility preparedness for outpatient management of hypertension diseases, Tanzania SPA 2014–15 (N = 725).

Of the 725 surveyed facilities, only 28% were found to be prepared to provide the outpatient primary care of hypertension (Fig 1)

### Availability of basic components for the outpatient primary care of hypertension

**Fig 2:**
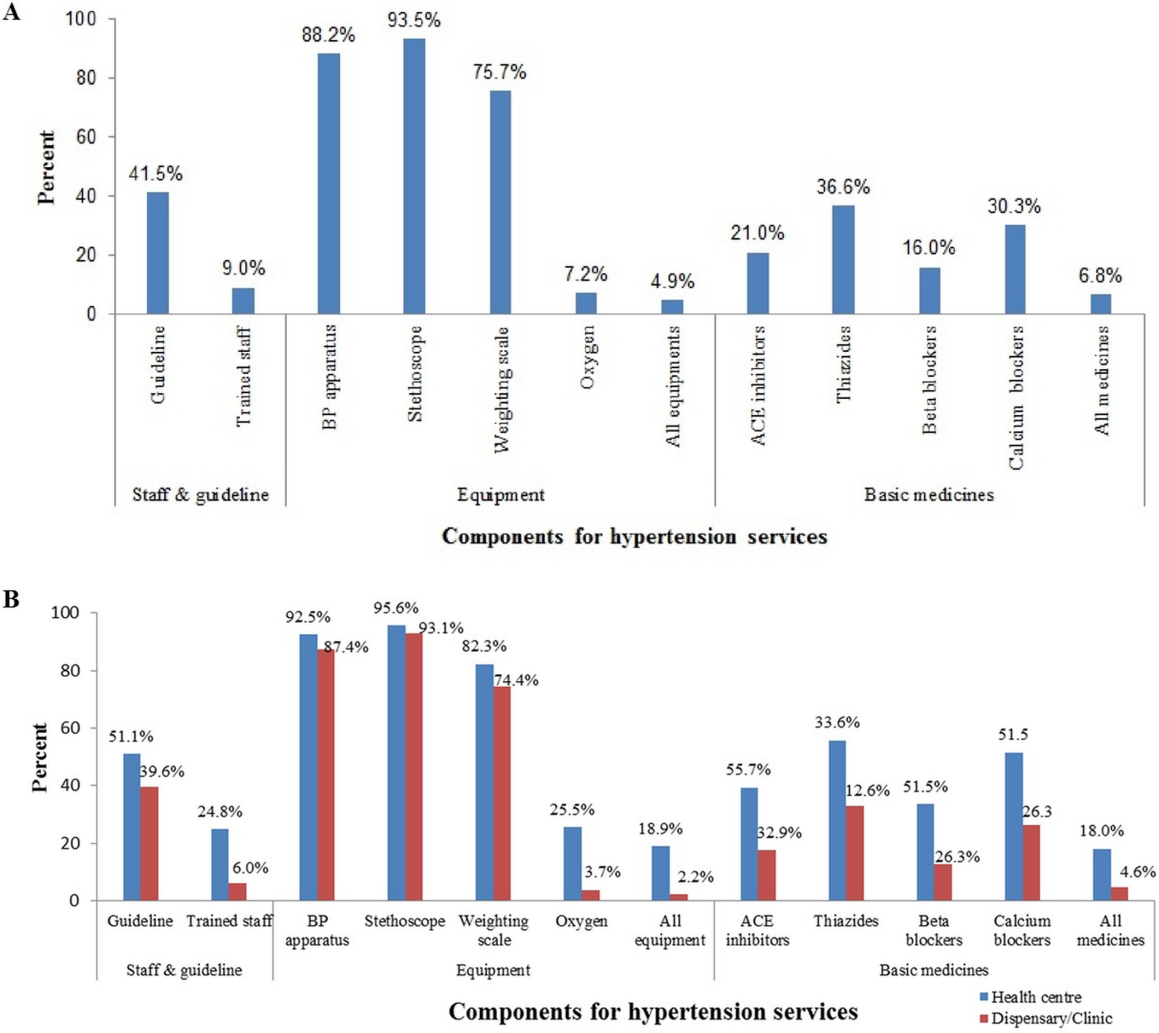
Service availability related to hypertation care, Tanzania SPA 2014–15 (N = 725). (A) Availability of basic components that support provision of outpatient primary care for hypertension among lower-level facilities and (B) Availability of basic components that support provision of outpatient primary care for Hypertension among lower-level facilities by type of facility.

Out of the 725 surveyed facilities, 42% had HTN guidelines and 9% had at least one staff that has undergone HTN care training. Only 5% of the facilities had all the equipment highlighted by WHO for the provision of HTN services. Regarding availability of basic anti-hypertensive medications, 21% of the facilities had ACE inhibitors, 37% had thiazides diuretics and 16% had beta-blockers, but only 7% of the facilities had all basic medicines Fig 2A.

### Preparedness for the outpatient primary care of hypertension by facility type

When we contrasted health centers and dispensaries/clinics, availability of diagnostic equipment was differ according to type of facility (BP apparatus (93% vs 87%), stethoscope (96% vs 93%), and weighing scale (82% vs 74)). Regarding NCD guidelines and trained staff, there were 51% of health centers and 40% of dispensaries with available NCD guideline and 25% vs 6% with trained staff, respectively. Availability of first-line medication therapy was relatively higher in health centers than dispensaries (ACE-Inhibitors (39% vs 18%), thiazides diuretics (56% vs 33%), beta-blockers (34% vs 13%), and calcium channel blockers (52% vs 26%)) Fig 2B.

### Factors associated with facility preparedness for the outpatient primary care of Hypertension

In multivariate analysis, privately-owned facilities [AOR = 2.7, 95% CI; 1.2–6.1], urban location [AOR = 2.2, 95% CI; 1.2–4.2], type of facility [AOR = 5.2, 95% CI; 3.1–8.7] (health center compared to clinic/dispensary), presence of routine management meetings [AOR = 2.6, 95% CI; 1.1–5.9] were significantly associated with being prepared to provide the outpatient primary care of HTN, Table 2.

**Table 2:** Factors associated with preparedness for outpatient management of hypertension diseases, adjusted for confounding variables, Tanzania SPA 2014–15 (N = 725).

Variable	COR [95% CI]	AOR [95% CI]
**Managing authority** (ref: Public)		
Private	3.9 [2.4–6.5]	2.7 [1.2–6.1]*
**Facility type** (ref: Clinic & dispensary)		
Health Centre	4.9 [3.5–7.5]	5.2 [3.1–8.7]**
**Facility Location** (ref: Rural)		
Urban	4.2 [2.7–6.6]	2.2 [1.2–4.2]*
**Routine management meetings** (ref: No)		
Yes	3.2 [1.6–6.4]	2.6 [1.1–5.9]*
**External supervision** (ref: Not received)		
Received	0.8 [0.2–3.9]	—
**External source of revenue** (ref: government)		
Other than government	2.1 [1.3–3.4]	1.3 [0.7–2.6]
None	2.9 [1.4–6.2]	1.1 [0.4–3.3]
**User fees** (ref: fixed for all services)		
Separate fee	2.6 [1.6–4.1]	1.2 [0.6–2.4]
**Health insurance** (ref: Not accepted)		
Accepted	0.8 [0.5–1.3]	—

Note:

* = P-value < 0.05

** = P-value < 0.001,—not included in multivariate analysis

## Discussion

The current study aimed to assess the preparedness of lower-level health facilities for the outpatient primary care of HTN and its associated factors assessed by using data from Tanzania national survey. The study found that a small proportion of lower-level health facilities were prepared for the outpatient primary care of HTN even though the majority had reported offering HTN services. Also, it found that availability of trained staffs, relevant guidelines, basic diagnostic equipment, and first-line medicines was very minimal in these facilities. Furthermore, the study found that the urban location, privately owned facilities, level of the facility (health center) and performing routine management meetings were significantly associated with being prepared to offer the outpatient primary care for HTN.

According to the Tanzania national health policy, lower-level health facilities are expected to manage all uncomplicated diseases or conditions such as HTN and stabilize some of the complicated symptoms as outpatient cases before referring them to higher-level facilities (hospitals) [34]. However, the current study found a low proportion of lower-level health facilities were prepared to offer adequate care services for HTN. This finding highlights the needs for strengthening lower-level facilities so as to cope with the rising burden of HTN and other NCDs. The low proportion of preparedness found in this study is in agreement with findings from previous studies conducted in northwestern Tanzania and Uganda among a few selected health facilities (25,26,30). The similarity in findings is likely due to comparable geographic characteristics and implementation of related health programs since these countries have similar socio-economic determinants.

The availability of guidelines and staff training are crucial to provide the knowledge and skills required to improve healthcare processes and patient outcomes [35,36]. Evidence from several studies shows that training primary health care providers may provide a solution in the management of HTN and other NCDs [37,38]. However, our study found a low proportion of lower-level health facilities in Tanzania with both guidelines and at least one staff member who has received training about HTN services. A similar finding has been reported in other studies done elsewhere [28,39,40].

The current study found that the majority of lower-level health facilities in Tanzania were well-equipped in terms of basic equipment for diagnosis and monitoring of HTN (BP apparatus, stethoscope and weighing scale) when each equipment was assessed separately. More than a half of facilities were found to have stethoscopes, BP machines or weighing scales; however, the presence of a combination of all equipment was suboptimal. This study observed that these facilities were also inadequately equipped in terms of availability of essential first-line drugs for HTN management. This finding is consistent with findings reported by other studies [27,41,42].

The current study found the difference in overall availability of diagnostic equipment and essential medicines for the management of HTN according to the type of facility. Health centers reported a higher availability of diagnostic equipment and basic medicines than dispensaries. This observed difference might be explained by the lack of clear formula at the district level, on how to allocate funds between health centres and dispensaries that may contribute to inefficiencies and inequities in the distribution of medicines and equipments [43]. Similar findings have been reported by previous studies conducted in other developing countries [27,28,39,44].

In low and middle-income countries, private health facilities are argued to be more efficient, accountable, and sustainable in healthcare delivery compared to public facilities [45,46]. In agreement with this, the current study found that the odds of preparedness for the outpatient primary care of HTN were higher among private sector facilities compared to public facilities. This finding might be explained due to the fact that lower-level public facilities follow a complex ordering process for basic medicines and equipment that needs permission and approval from District Medical Officer (DMO), therefore, this long chain might be a result of low availability of these items in public facilities [47,48].

Previous evidence shows significant differences in availability and provision of health care between urban and rural health facilities [44,47,49]. This is supported by the current study, which found that the odds of preparedness for outpatient HTN treatment are higher among urban health facilities compared to rural health facilities. This finding might be due to the fact that some of the health facilities were located in the hard to reach rural areas with, resulting to inefficiecncies in managing supply logistic systems that lead to the low availability of basic medicines and equipment [50].

As expected, the current study found the difference in preparedness according to the level and type of facility. The study revealed that the odds of preparedness are higher among health centers compared to dispensaries. However, the current study contrasts with the finding from the previous study conducted in Dar-es-Salaam, Tanzania [51]. The difference of the findings between the current and the previous one might be due to study setting. The previous study evaluated one region and specifically concentrated on HIV care and treatment clinics, while the current study used data collected from the nationwide sample and involved all type of health facilities.

To cope with the rising burden of HTN and other NCDs, there is a need to strengthen primary health care systems in Tanzania. Several studies have highlighted the importance of prioritizing primary health care for the management of NCDs [52,53]. Prevention, control, and management of NCDs should start at the primary health care level, where the majority of the population is served. A study done in Uganda concluded that strengthening NCD care at the primary health care level would not have a significant budget impact [54]. Improving the availability of diagnostic equipment, first line drugs, recommended guidelines and trained staff, which have been shown to be the main challenges for HTN and other NCD services in our study and the study in northwestern Tanzania [27], could improve the provision of these services.

The strength of the present study is that it was the first analysis to assess the preparedness of lower-level health facilities for the outpatient primary care of HTN in this region by using the TSPA 2014–2015 dataset, which is a nationwide, representative sample of health facilities, with a response rate of 99%. The use of such a comprehensive national data set suggests that the present findings accurately reflect the current situation of the outpatient primary care for HTN in Tanzania. Also, by considering the complex sampling techniques involved, we adjusted our findings for clustering effect and weighted the analysis to correct for non- response and disproportionate sampling. This study assesses the provision of HTN services, which has been shown to be the most prevalent NCD in Tanzania and used it as a representative point to assess the preparedness of health systems, which has been used elsewhere [55].

However, the current study has some limitations, due to the weakness of cross-sectional survey, it fails to explain the causality assumptions, therefore, the results should be interpreted with caution. The study restricted to lower-level facilities that reported to offer HTN services, this might affect the statistics found in this study as it excluded hospital and facilities reported not to offer any HTN services. Using arbitrary cut off point set at 50% may misclassify preparedness of facilities for the outpatient primary care of HTN.

## Conclusion

The primary health care system in Tanzania is not adequately equipped to successfully manage HTN and other NCDs. The proportion of lower-level health facilities being prepared for the outpatient primary care of HTN was low. Rural location, public ownership, and absence of routine management meetings were associated with being not prepared. There is a need to strengthen the primary health care system in Tanzania to cope with the increasing burden of HTN and other NCDs. Key focus areas should be: increasing the availability of HTN guidelines, provision of refresh training courses about HTN and other NCDs, increasing the availability of basic diagnostic equipment for the major NCDs such as HTN, and basic drugs, especially in rural and public facilities.
